# In-depth characterization of T cell responses with a combined Activation-Induced Marker (AIM) and Intracellular Cytokine Staining (ICS) assay

**DOI:** 10.1093/oxfimm/iqae014

**Published:** 2024-12-09

**Authors:** Yeji Lee, Alison Tarke, Alba Grifoni

**Affiliations:** Center for Vaccine Innovation, La Jolla Institute for Immunology (LJI), La Jolla, CA 92037, United States; Center for Vaccine Innovation, La Jolla Institute for Immunology (LJI), La Jolla, CA 92037, United States; Center for Vaccine Innovation, La Jolla Institute for Immunology (LJI), La Jolla, CA 92037, United States

**Keywords:** T cell, AIM, ICS, CD107a

## Abstract

Since T cells are key mediators in the adaptive immune system, evaluating antigen-specific T cell immune responses is pivotal to understanding immune function. Commonly used methods for measuring T cell responses include Activation-Induced Marker (AIM) assays and Intracellular Cytokine Staining (ICS). However, combining these approaches has rarely been reported. This study describes a combined AIM + ICS assay and the effect of collecting the supernatant. Peripheral blood mononuclear cells (PBMCs) from seven healthy donors were stimulated with DMSO (negative control), Epstein-Barr virus (EBV) peptide pools, and PHA (positive control). The AIM markers OX40 + CD137+ were used for CD4+ T cells and CD69 + CD137+ and CD107a + CD137+ for CD8+ T cells. Cytokine-secreting cells were identified as CD40L+ cytokine+ for CD4+ and CD69+ or CD107 + cytokine+ for CD8+ T cells. Half of the supernatant was collected before adding the BFA/Monensin/CD137 antibody solution to assess the impact on T cell responses. The CD107a + CD137+ AIM markers combination had a lower background than CD69 + CD137+, making CD107a+ a more sensitive marker for CD8+ AIM markers. Collecting half of the supernatant did not significantly affect the immune responses. Our AIM + ICS combined protocol enables the simultaneous assessment of activation and cytokine release reducing the sample volume for testing T cell responses. We also show that collecting half of the supernatant does not significantly interfere with immune responses detection.

## Introduction

T cells are critical mediators of the adaptive immune system. Investigating antigen-specific T cells is essential for understanding immune responses [1]. Many methods have been developed to measure antigen-specific immune responses, such as surface marker staining, peptide-human leukocyte antigens (HLA)-specific multimer staining, cell proliferation assays, and measuring cytokines secreted from the cells [2]. Various surface markers for activated T cells have been identified, including OX40 (CD134), 4-1BB (CD137), CD25 (IL-2 receptor alpha), and CD69 [3]. Upon T cell activation, the expression of 4-1BB, CD25, and CD69 increases and peaks at 24-, 48-, and 16-h post-stimulation, respectively. Unlike other activation markers, OX40 is not expressed on resting T cells, with its expression peaking 16 h after activation [3]. In previous studies, our group demonstrated the use of OX40 + 4-1BB+ for CD4+ T cells and CD69 + 4-1BB+ for CD8+ T cells as Activation-Induced Markers (AIM) to measure antigen-specific CD4+ and CD8+ T responses against peptide pools [4–7]. The AIM assay identifies antigen-specific activated T cells by the upregulation of different combinations of activation markers after antigen stimulation. An advantage of the AIM assay is that it has been used to identify T cells that secrete a limited amount of cytokines, such as follicular helper T cells, by staining with PD-1 and CXCR5 in addition to the activation markers [7]. However, the AIM assay alone cannot elucidate cytokine profiles or transcription factor expression, which requires further downstream assays [8].

Intracellular cytokine staining (ICS) measures the accumulation of cytokines produced by T cells after antigen stimulation. Other methods of measuring cytokine production, such as Enzyme-Linked ImmunoSorbent Assay (ELISA) or Enzyme-Linked Immunosorbent Spot (ELISpot), measure only the cytokines secreted from whole peripheral blood mononuclear cells (PBMCs; or the immune cells plated); with these methods it is hard to conclude the origin of cytokines among immune cells. Unlike other methods, the benefit of the ICS assay is the simultaneous identification of the specific immune cells producing the cytokines by combining the intracellular staining with the staining of membrane markers, such as CD4 or CD8 to help diversifying the specific immune population [9]. However, several immune populations recognizing the antigen are poorly cytokine producers, such as the case of Tfh [7]. Therefore, examining AIM or ICS alone limits information compared to performing combined assays, which allows us to explore the cell source of cytokines and the functionality of activated T cells.

Another functional capability of T cells, in addition to cytokine production, is the release of cytotoxic granules and cell killing. To measure this functionality, previous work has focused on staining the membrane surface marker CD107a (LAMP-1) as a proxy marker for degranulation in activated T cells. Cytotoxic T cells kill target cells through two main pathways: one is the Perforin-Granzyme-mediated apoptosis, and the other one is the Fas-Fas ligand-mediated apoptosis [10]. In perforin-granzyme-mediated apoptosis, pre-formed cytotoxic granules inside cells move to the cell membrane, merge with the cell membrane, and degranulate. When degranulated, CD107a from the granule membrane can be detected by the anti-CD107a antibodies [11]. Thus, CD107a expression is associated with activation/degranulation of T cells [12]. Here, we compared the activation markers CD69 + CD137+ and CD107a + CD137+, as well as CD69 + cytokine+ and CD107a + cytokine+, to determine the most suitable markers for assessing T cell responses for future viral indications.

We also investigated the impact on T cell responses of collecting the assay culture supernatants prior to adding a solution of Brefeldin A (BFA), Monensin, and anti-CD137 antibody. Collecting the culture supernatants also offers an opportunity to measure secreted cytokines using additional detection assays, such as ELISA or Cytometric Beads Array (CBA) assays.

## Protocol: Activation-Induced Marker (AIM) assay in combination with Intracellular Cytokine Staining (ICS) and supernatant collection

This protocol was developed in order to address the need for an in-depth T cell characterization assay that could simultaneously determine (i) the type of T cell responding, (ii) the activation of the T cell, (iii) the cytokines being produced, and (iv) additionally allow for the collection of supernatants for downstream analysis of other cytokines or chemokines being releases. A core advantage of this assay is that it yields a high quantity and quality of data from relatively small amounts of cells, which is crucial when working with limited cell numbers, especially with acute patient samples or samples from endemic areas. Herein, we describe how to acquire cytokine-secreting T cells as well as activated T cells responding to viral peptides. In brief, PBMCs were plated at 1 × 10^6^ cells per well and stimulated with DMSO as a negative control in triplicate, EBV peptide pools, or phytohemagglutinin (PHA) as a positive control for 20 h at 37°C. Here, we show the T cell responses after removing the supernatant before adding the BFA, Monensin, and anti-CD137 antibodies and not removing the supernatant, to understand if removing the supernatant affects the results. After an additional 4-h incubation, the cells were stained with membrane antibody cocktails to identify activated T cells. The cells were then fixed with 4% paraformaldehyde (PFA) and stored at 4°C until the following day when the cells were permeabilized with saponin buffer. Then the cells were treated with blocking buffer before intracellular staining with antibodies followed by acquisition on by flow cytometer.

## Materials

RPMI 1640 (Corning #10–041-CM)

Heat-inactivated human serum AB (GeminiBio, #100–512)

Penicillin/streptomycin (GeminiBio, #400-109)

L-glutamine (GlutaMAX; Gibco, #35050-061)

Cryopreserved PBMCs

Benzonase (EMD Millipore Corp, #70664-3)

Dimethylsulfoxide (DMSO; Sigma, #D2650)

Phytohaemagglutinin (PHA; Roche, #431784-5MG)

EBV megapool (15-mer peptides encompassing EBV protein were used in this protocol.)

Phosphate-buffered saline (PBS; GIBCO BRL, #10010-023)

Saponin powder (Sigma, #47036)

10% bovine serum albumin (BSA; Sigma, #A-3294. Store at 4°C)

1% sodium azide (Sigma, #S2002; store at 4°C)

Conical polypropylene tubes (Corning Falcon #352070)

37°C water bath

Tabletop centrifuge

4% Paraformaldehyde (PFA) in PBS (Sigma #P6148)

Brilliant Staining Buffer Plus (BD, #566385)

96-well U-bottom cell culture-treated plate (GenClone, #25–221)

Flow cytometer (Aurora Spectral flow cytometer (Cytek) was used in this protocol.)

## Reagents

HR5 medium (1000 ml RPMI 1640 with 5% of heat-inactivated human serum AB, 1% of penicillin/streptomycin, and 1% of L-glutamine).

*NOTE*: The volumes of antibodies used in this protocol were determined based on antibody titration. Antibody volumes can be different depending on the sensitivity of the flow cytometer machine.

## Human samples

PBMCs were purchased as a fee-for-service from a company as de-identified biospecimens and therefore do not meet the definition of human subject research. Donor characteristics are shown in Table 1.

**Table 1.** Donor characteristics

**Total subjects**	*n* = 7
**Age (years; IQR)**	28.0 [25.0–380]
**Gender (% female)**	28.6
**Ethnicity (%)**	
White	71.4
American Indian/Alaska Native	14.3
Unknown or not reported	14.3

## Peptide megapool preparation

The megapool (MP) approach and preparation have previously been described in detail [13]. In brief, we synthesized peptides as crude material (TC Peptide Lab, San Diego, CA) based on experimentally defined EBV data to make two MPs optimized for CD4 and CD8 T cells as previously described [14, 15]. To make the CD4 and CD8 EBV MPs, the individual peptides were resuspended in DMSO at a concentration of 10 mg/ml, and aliquots were pooled before performing a sequential lyophilization. Each lyocake was subsequently resuspended in DMSO at 1 mg/ml, as previously described [5, 16].

## Day 1

### Thawing of PBMCs

1. Prepare sterile 15 ml conical tubes. For each vial of PBMCs to be thawed, add 10 ml of HR5 medium and 20 μl of Benzonase.
2. Thaw vial(s) of PBMCs by placing the vials in a 37°C water bath for 45 s or until the edges of the ice pellet have just begun to thaw.
3. Quickly transfer the cells into the 15 ml tubes and centrifuge the tubes for 7 min at 1200 rpm at 4°C. Discard the supernatant.
4. Resuspend cells in 10 ml of HR5 medium and count cell numbers.
5. Centrifuge the tubes for 7 min at 1200 rpm at 4°C. Discard the supernatant.
6. Add HR5 to bring the PBMCs to a concentration of 20 × 10^6^/ml and plate 50 μl of 1 × 10^6^ PBMCs in each well of 96-well U-bottom cell culture-treated plates.

### Cell stimulation

7. Prepare stimuli solutions according to Table 2.
8. Add 50 μl of appropriate stimuli solution to each well.
9. Prepare CD107a solution according to Table 3.
10. Add 50 μl of CD107a solution to each well for a total of 150 μl.
11. Incubate plates for 20 h at 37°C in 5% CO_2_.

**Table 2.** Stimuli solutions

Stimuli	Stock	4× final (μg/ml)	V stimuli (μl)	HR5 (μl)	Final assay (μg/ml)
DMSO (triplicate)	100%	4	2	500	1
EBV MPs (CD4 + CD8)	1 mg/ml	4	2 + 2	500	1
PHA	1 mg/ml	4	2	500	1

**Table 3.** Antibody cocktails to add in culture

Solution	Reagent/antibody	Fluorochrome	Clone/source/catalog number	Amount (μl)/well
CD107a solution	CD107a	BB700	HYA3/BD/566558	0.6
	HR5 medium	–	–	50
BFA/Monensin/CD137 solution	BFA (Golgi Plug)	–	BD/555029	0.2
	Monensin (Golgi Stop)	–	BD/554724	0.2
	CD137	APC	4B4-1/Biolegend/309810	1
	HR5 medium	–	–	50

## Day 2

### Adding BFA, Monensin, and anti-CD137 antibody solution

12. Collect half (75 μl) of the supernatant from each well and either freeze at −20°C for future analysis.
13. Replenish the media by adding 75 μl of HR5 with stimuli solutions according to Table 2.
14. Prepare HR5 medium containing BFA, Monensin, and anti-human CD137 antibody according to Table 3.
15. Add 50 μl of the BFA/Monensin/CD137 solution per well and resuspend cells.
16. Incubate for an additional 4 h at 37°C in 5% CO_2_.

### Antibody membrane staining

17. Prepare membrane staining antibody cocktail according to Table 4.
18. After 4 h, centrifuge the plates for 3 min at 1400 rpm at 4°C. Discard the supernatant.
19. Add 150 μl of PBS per well and centrifuge the plates for 3 min at 1400 rpm at 4°C. Discard the supernatant.
20. Add 100 μlL of prepared Membrane Staining Cocktail to the wells and incubate for 30 min at 4°C in the dark.
  - Remember to take out 4% PFA solution from the −20°C freezer to allow it to thaw at room temperature.
21. After 30 min, add 100 μl of PBS and centrifuge the plates for 2 min at 1400 rpm at 4°C. Discard the supernatant.
22. Wash the plates with 150 μl of PBS and centrifuge the plates for 2 min at 1400 rpm at 4°C. Discard the supernatant.

**Table 4.** Antibody cocktails for staining

Cocktails	Reagent/antibody	Fluorochrome	Clone/source/catalog number	Amount (μl)/well
Membrane staining cocktail	LIVE/DEAD	APC-eFluor780	ThermoFisher/L34976	0.02
	OX40	PE-Cy7	Ber-ACT35/Biolegend/350012	0.25
	CD14	APC-eFluor780	61D3/ThermoFisher/47–0149-42	0.25
	CD19	APC-eFluor780	HIB19/ThermoFisher/47–0199-42	0.25
	CD69	BV786	FN50/BD/563834	0.5
	CD3	BUV805	UCHT1/BD/612895	1
	CD8	BUV496	RPA-T8/BD/612942	1
	CD137	APC	4B4-1/Biolegend/309810	1
	CD4	BV605	RPA-T4/BD/562658	1.5
	Brilliant staining buffer plus	–	–	10
	PBS	–	–	90
Intracellular staining cocktail	IFNg	FITC	4S.B2/ThermoFisher/11–7319-82	0.25
	TNFa	eFluor 450	MAb11/ThermoFisher/48–7349-42	0.5
	Perforin	Alexa Fluor 700	B-D48/Biolegend/353324	0.5
	IL-4	BUV737	MP4-25D2/BD/612835	0.5
	CD40 Ligand	PE	TRAP1/BD/555700	1
	IL-10	PE-Dazzle594	JE53-19F1/Biolegend/506812	1.5
	Saponin buffer	–	–	50

### Fixation

23. Add 150 μl of 4% PFA to each well, resuspend cells, and incubate for 10 min at 4°C in the dark.
24. Centrifuge the plates for 5 min at 2000 rpm at 4°C. Discard the supernatant.
25. Wash twice with 150 μl of PBS for 5 min at 2000 rpm at 4°C.
26. Proceed directly to Step 27, or add 150 μl MACS buffer to each well and store at 4°C in the dark overnight.

## Day 3

### Permeabilization and blocking

27. Prepare Saponin Buffer and Blocking Buffer according to Table 5. These buffers should be made fresh on the day of use.
28. Centrifuge the plates for 5 min at 2000 rpm at 4°C. Discard the supernatant.
29. Add 150 μl of Saponin Buffer per well and centrifuge the plates for 5 min at 2000 rpm at 4°C.
30. Add 50 μl of Blocking Buffer to block all wells and incubate for 15 min at room temperature in the dark.

**Table 5.** Permeabilization and blocking buffers

Buffers	Reagents	No. of wells	
		1	100
**Saponin Buffer**	Saponin powder	0.002 g	0.2 g
	10% BSA	0.04 ml	4 ml
	1% sodium azide	4 μl	400 μl
	PBS	0.36 ml	36 ml
	Final Volume	0.4 ml	40 ml
**Blocking Buffer**	100% Human serum AB	6 μl	600 μl
	Saponin buffer	0.054 ml	5.4 ml
	Final volume	0.06 ml	6 ml

### Intracellular staining

31. Prepare an intracellular staining cocktail according to Table 4.
32. Add 50 μl intracellular staining cocktail to all wells. Incubate for 30 min at room temperature in the dark.
33. Centrifuge plates for 5 min at 2000 rpm at 4°C. Discard the supernatant.
34. Add 150 μl of Saponin buffer per well and centrifuge for 5 min at 2000 rpm at 4°C. Discard the supernatant.
35. Add 150 μl of PBS per well and centrifuge the plates for 5 min at 2000 rpm at 4°C. Discard the supernatant.
36. Resuspend cells in 100 μl of PBS.
37. Wrap the plates in aluminum foil and store them at 4°C until ready to acquire on the flow cytometer.

## Interpreting the data

**Figure 1.**
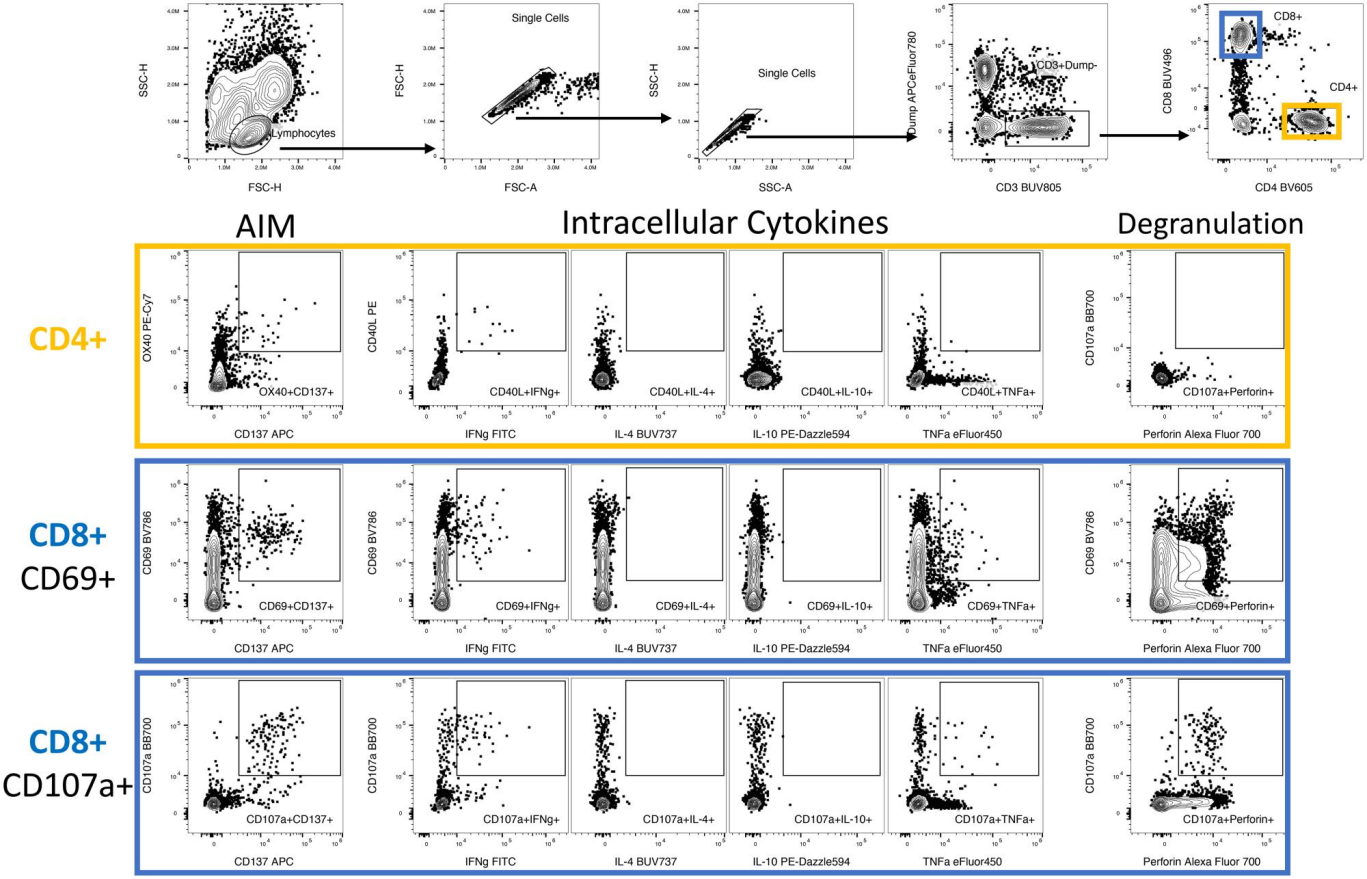
Gating strategy. The AIM + ICS assay gating strategy is outlined for each antibody used in this protocol. EBV-specific T cell responses are shown for both CD4+ and CD8+ T cells for a representative donor. The gates are shown for AIM, cytokines, and degranulation markers.

The T cell responses to EBV MPs from 7 healthy donors were evaluated by AIM + ICS assay, and all samples were acquired using a Cytek Aurora Spectral Flow Cytometer. Data was analyzed with FlowJo software (Tree Star Inc.) and the gating strategy used is outlined in Fig. 1. In brief, lymphocytes were first gated, followed by two sequential single-cell gates to exclude duplicate cells. The CD3+ gate was drawn to exclude dead cells, CD14+, and CD19+ cells with the dump channel. CD4 + AIM+ T cells were further defined by AIM markers as OX40 + CD137 (4-1BB)+, and CD8 AIM+ T cells were defined as CD69 + CD137+.

For ICS, cytokine-producing CD4+ T cells were defined as CD40L + cytokine+ CD4+ T cells, and cytokine-producing CD8+ T cells were defined as CD69 + cytokine+ CD8 + T cells for each cytokine (IFNg, IL-4, IL-10, Perforin, and TNFα). For CD8+ T cells, we explored an additional combination of markers, using CD107a instead of CD69 in combination with CD137+ to measure the activated cells and also to measure CD107a + cytokine+ CD8+ T cells, as shown in Fig. 1.

The resulting data from this gating strategy was refined by removing the background signal by subtracting the average of the percent of AIM+ or ICS+ for the triplicate DMSO-stimulated controls. The average of the DMSO controls was also used to calculate the Stimulation Index (SI) by dividing the percent of AIM+ or ICS+ cells after EBV MP stimulation with the average percent in the negative DMSO control. The Limit of Sensitivity (LOS) was defined as the median plus two standard deviations of the negative DMSO controls. In contrast, the Limit of Detection (LOD) was defined as two times with a 95% upper confidence interval of the geometric mean of the negative DMSO controls. A response was considered positive only if the background subtracted value was greater than the LOS and at least two times greater than the SI. The LOS and LOD were calculated for each combination of AIM markers, while instead, the LOS and LOD were calculated based on CD40L + IFNg+ for CD4+ T cell cytokine responses and CD69 + IFNg+ or CD107a + IFNg+for CD8+ T cell cytokine responses.

## Results

To identify appropriate markers to capture Perforin-secreting T cells, we first compared CD40L + Perforin+ and CD107a + Perforin+ for CD4+ T cells, and CD69 + Perforin+ and CD107a + Perforin+ for CD8+ T cells (Supplementary Fig. 1). Representative plots of DMSO-stimulated cells show a very low background signal for Perforin + CD4 + T cells with both CD40L or CD107a (Supplementary Fig. 1A). For DMSO-stimulated CD8+ T cells, the CD69 + Perforin+ cells have a high background compared to the DMSO-stimulated CD107a + Perforin+CD8+ T cells (Supplementary Fig. 1B). Within CD4+ T cells, the magnitude of response to EBV MPs is significantly higher than the negative control for CD40L + Perforin+ (Wilcoxon *P* = .002) while there is no difference in the CD107a + Perforin+ populations (Wilcoxon *P* = .625, Supplementary Fig. 1C). For CD8+ T cells, the high background signal with CD69 + Perforin+ makes it difficult to measure the EBV-specific response compared to the negative control (Wilcoxon *P* = .1189). Instead, CD107a + Perforin+ shows a clear and significant EBV-specific CD8+ T cell response compared to the DMSO negative control (Wilcoxon *P* = .0166, Supplementary Fig. 1D). That indicates that CD107a is a more sensitive and discerning marker for measuring Perforin + T cell responses.

**Figure 2.**
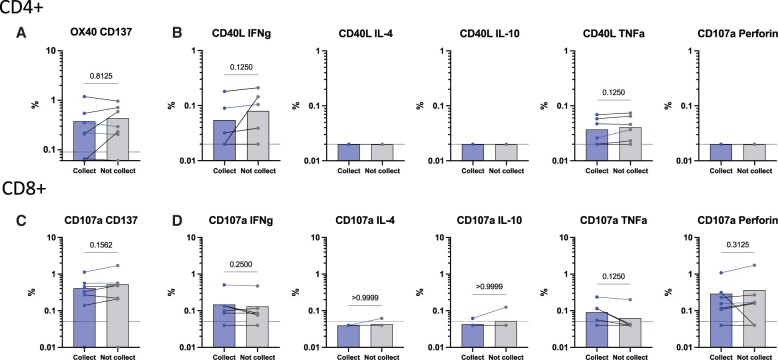
Comparison of T cell responses after collecting or not collecting supernatants. EBV-specific AIM and ICS T cell responses are shown for PBMCs from healthy donors (*n* = 7). Both CD4+ and CD8+ T cell responses are shown after collecting supernatants (Collect = blue bars) or not collecting supernatants (Not collect = grey bars). EBV-specific CD4+ T cell responses are shown for AIM OX40 + CD137+ (A) and for ICS (B) IFNg, IL-4, IL-10, TNFa, and Perforin in combination with CD40L. EBV-specific CD8+ T cells responses are shown for AIM CD107a + CD137+ (C) and for ICS (D) IFNg, IL-4, IL-10, TNFa, and Perforin in combination with CD107a. The dotted line indicates the LOS. Bars represent the mean, and all data is subtracted from the background and SI > 2. *p* values listed on the top of each graph correspond to a Wilcoxon matched-pairs signed rank test.

Next, we evaluated whether collecting half of the supernatant before adding BFA, Monensin, and anti-CD137 antibodies solution could affect the T cell responses. Since BFA and Monensin prevent cytokines from being secreted outside of cells, we had collected half of the supernatant before the BFA and Monensin were added so that the cytokines secreted in the first 20 h of stimulation could be analyzed in the future from these supernatants. Our results show that collecting half of the supernatant has no significant effect on the AIM + responses (Fig. 2A, Wilcoxon *t*-test, *P* = .8125) nor the cytokine responses in CD4+ T cells (Fig. 2B, Wilcoxon *t*-test, IFNg and TNFa *P* = .125). Similarly, the CD8+ T cell response was not impacted by the collection of half of the supernatants in the AIM + markers (Fig. 2C, Wilcoxon *t*-test, *P* = .1562) or ICS (Fig. 2D, Wilcoxon *t*-test, IFNg *P* = .25, IL-4 *P* > .9999, IL-10 *P* > .9999, TNFa *P* = .125, and Perforin *P* = .3125) markers. The option to remove half of the supernatants before adding BFA and Monensin allows us to explore an opportunity to measure cytokines secreting from the cells in downstream applications either as a part of the assay or independently focusing on specific ag-specific T cell combinations selected after the AIM/ICS data analysis.

## Critical parameters

The AIM + ICS assay described herein uses live PBMCs, and it is essential that they are viable and functional at the time of the assay. When using frozen PBMCs, it is necessary to ensure that the cryopreserved vials have been properly stored and thawed to maximize cell viability as well as recovery. Deviating from the protocol outlined here could compromise the viability of the cells and the results of the assay. For instance, when thawing the PBMCs, the vial should be removed from the water bath long enough to melt the ice completely. The contents of the vial should be diluted in HR5 as soon as it has begun to thaw in order to minimize exposure to the high concentration of DMSO in the freezing medium.

Previously, we had determined the effect of added BFA and Monensin in the culture on the expression levels of the AIM markers used in this assay. In this way, we adjusted the protocol from the previously described AIM assays to include CD137 for the last 4 h of stimulation when the BFA and Monensin were added to the culture in order to counter the lower expression of this marker that we noticed with the addition of the blocking reagents [17]. It is important to note that the specific CD69 and OX40 antibodies used in this protocol were not affected by the addition of BFA and Monensin for this specific set of marker combinations. In other indications, additional markers have been included in this combined protocol to retain comparable surface marker expression. It is accordingly critical to carefully determine what markers are affected by downregulation based on the panel used and ensure to include those for the last 4 h to circumvent a decrease in expression levels following BFA and Monensin.

Similarly, it is necessary to add the CD107a antibody at the beginning of the culture on Day 1. This will account for the kinetics of the CD8 T cell response to the stimuli and will allow detection of degranulation throughout the 24-h incubation, as previously reported [12, 17].

All antibodies used in this protocol should be properly titrated for the specific flow cytometer and settings with which the cells will be acquired. The antibodies described here have been titrated to the optimal volume for the Cytek Aurora cytometer.

It is important to make the Saponin and Blocking Buffers fresh on the day of use to ensure proper cell permeabilization. Altering this step can result in sub-optimal permeabilization and a lower signal for the intracellular antibodies.

## Challenges and limitations

One challenge to this assay is that it requires a time commitment of approximately 3 days. Day 1 is the assay set up and the start of the 20 h of incubation (approximately 1–3 h hands-on, depending on the size of the experiment), Day 2 is when the BFA/Monensin/CD137 antibodies solution is added for an additional 4 h followed by the membrane staining and fixation (hands-on approximately 2–3 h), and Day 3 is when the permeabilization and intracellular staining are done (hands-on approximately 2 h) followed by acquisition by flow cytometer. If desired, the permeabilization and intracellular staining can also be done on Day 2.

The number of cells required for this assay is a minimum of 1 × 10^6^ per condition. When testing ex-vivo samples, we do not recommend going below 1 × 10^6^ per condition, as you can have an artificially low signal due to the low precursor frequency of some antigen-specific T cells.

A limitation of this assay is that the membrane staining is done in PBS, which could allow for non-specific antibody binding and increased background signal. We plan to further optimize this protocol by performing this step in MACS Buffer (PBS containing BSA) with or without an Fc blocking reagent to prevent non-specific antibody binding to the Fc receptor.

## Supplementary Material

iqae014_Supplementary_Data

## Data Availability

The data underlying this article are available in the article and in its online supplementary material.
