# Supplementary material for: In-depth characterization of T cell responses with a combined Activation-Induced Marker (AIM) and Intracellular Cytokine Staining (ICS) assay
Source: Oxf Open Immunol. 2024 Dec 9;5(1):iqae014. doi: 10.1093/oxfimm/iqae014 (PMC11661976; doi:10.1093/oxfimm/iqae014)
Supplement: iqae014_Supplementary_Data [file iqae014_supplementary_data.docx]

**Supplementary Figure 1**

**Supplementary Figure 1. DMSO-stimulated backgrounds and comparison of DMSO-stimulated and EBV-stimulated T cells.** Representative plots of DMSO-stimulated CD40L+Perforin+ and CD107a+Perforin+ CD4+ T (**A**) and DMSO-stimulated CD69+Perforin+ and CD107a+Perforin+CD8+ T cells (**B**). DMSO-stimulated and EBV-stimulated CD40L+Perforin+ and CD107a+Perforin+ CD4+ T cells are shown in (**C**), and DMSO-stimulated and EBV-stimulated CD69+Perforin+ and CD107a+Perforin+ CD8+ T cells are shown in (**D**). Bars represent the mean, and *p* values listed on the top of each graph correspond to a Wilcoxon matched-pairs signed rank test.
